# Supplementary material for: ADAR1 Isoforms Regulate Let-7d Processing in Idiopathic Pulmonary Fibrosis
Source: Int J Mol Sci. 2022 Aug 12;23(16):9028. doi: 10.3390/ijms23169028 (PMC9409484; doi:10.3390/ijms23169028)
Supplement: Supplementary file 1 [file ijms-23-09028-s001.zip › Figure S1.pdf]

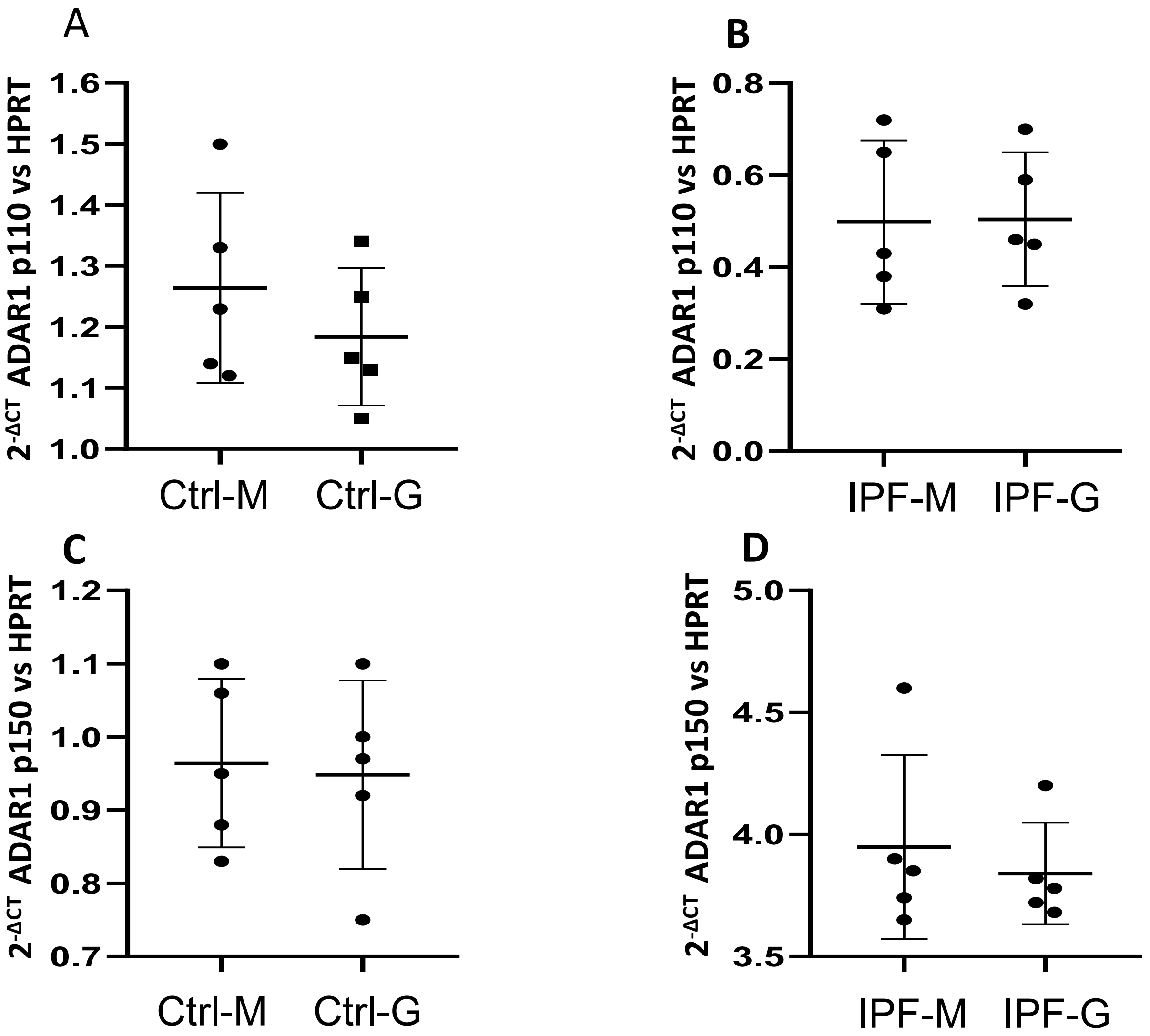

Figure S1. Basal expression of ADAR p110 and p150 in Fibroblasts Control and IPF in Mexican samples versus German samples. (A) ADAR1 p110 expression of Mexican control vs German control (p=0.3796), (B) ADAR1 p110 expression of Mexican IPF vs German IPF (p=0.9548), (C) ADAR1 p150 expression of Mexican control vs German control (p=0.9640), and (D) ADAR1 p150 expression of Mexican IPF vs German IPF (p=0.5905).
