# Supplementary material for: ADAR1 Isoforms Regulate Let-7d Processing in Idiopathic Pulmonary Fibrosis
Source: Int J Mol Sci. 2022 Aug 12;23(16):9028. doi: 10.3390/ijms23169028 (PMC9409484; doi:10.3390/ijms23169028)
Supplement: Supplementary file 1 [file ijms-23-09028-s001.zip › Figure S2.pdf]

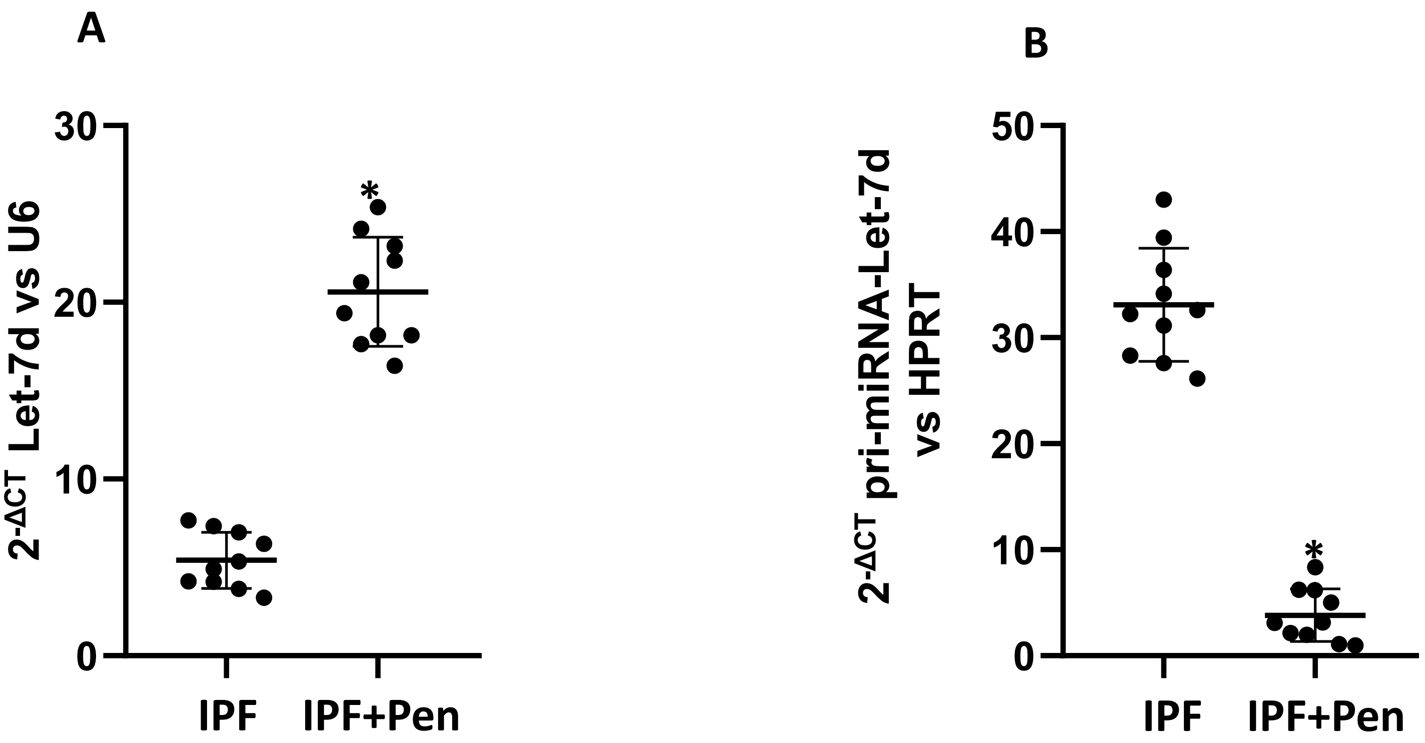

Figure S2. The expression of Let7d and pri-miR-Let7d on IPF fibroblasts is modified by Pentostatin (Pen) treatment.

In (A), IPF fibroblasts stimulated with Pentsotatin showed an increase of Let7d expression ($p<0.0001$); in (B), IPF fibroblasts stimulated with Pentostatin showed a decrease of pri-miR-Let7d expression ($p<0.0001$).
