# Supplementary material for: ADAR1 Isoforms Regulate Let-7d Processing in Idiopathic Pulmonary Fibrosis
Source: Int J Mol Sci. 2022 Aug 12;23(16):9028. doi: 10.3390/ijms23169028 (PMC9409484; doi:10.3390/ijms23169028)
Supplement: Supplementary file 1 [file ijms-23-09028-s001.zip › Table S1.pdf]

Table S1

**Clinical Characteristics**	**German Subjects**		**Mexican Subjects**	
	Control	IPF	Control	IPF
Age (years)%				
<50	60	20	60	-------------------
50-60	40	40	40	40
>60	--------------------	40	-------------------	60
FEV1/FVC %	--------------------	85 ± 3	-------------------	84.5 ± 5
Gender %				
Male	60	100	60	80
Female	40	--------------------	40	20
Smoking % history				
Former	40	-------------------	40	40
Never	60	--------------------	60	60
Total	5	5	5	5
