# Supplementary material for: ADAR1 Isoforms Regulate Let-7d Processing in Idiopathic Pulmonary Fibrosis
Source: Int J Mol Sci. 2022 Aug 12;23(16):9028. doi: 10.3390/ijms23169028 (PMC9409484; doi:10.3390/ijms23169028)
Supplement: Supplementary file 1 [file ijms-23-09028-s001.zip › Table S2.pdf]

qPCR primers		
**mRNA**	**Fwd primer**	**Rwd primer**
**ADAR1 p150**	CTTCCAGTGCGGAGTAGCG	ATTCATTGCGCCCGCGAG
**ADAR1 p110**	TGGCAGCCTCCGGGTG	TGTCTGTGCTCATAGCCTTG
**APOBEC3D wt**	GTCCAGGCTGGAATGCAATGTCA	GAGGCTGAAGCAGAAGAATCG CTTAAAC
**APOBEC3D edit**	CTCTGGGATCTCTCTGCCTCCAAA TATC	GAGGTTGCAGTGAGTCCAGAT GGC
**Cloning primers**		
***ADAR1* p110 clon**	AAGGATCCATGGCCGAGATCAA	CCTCTAGACTATACTGGGCAG AG
**TaqMan Assay (Thermo Fisher)**		
**pri-miRNA-Let-7d**	Hs03302562_pri	
**Let-7d**	4427975 ID 002283	
**RNU6B**	4427975 ID 001093	
**HPRT1**	Hs02800695_m1	
